# Computational analysis of probabilistic reversal learning deficits in male subjects with alcohol use disorder

**DOI:** 10.3389/fpsyt.2022.960238

**Published:** 2022-10-19

**Authors:** Başak Bağci, Selin Düsmez, Nabi Zorlu, Gökhan Bahtiyar, Serhan Isikli, Adem Bayrakci, Andreas Heinz, Daniel J. Schad, Miriam Sebold

**Affiliations:** ^1^Department of Psychiatry, Katip Celebi University Ataturk Education and Research Hospital, İzmir, Turkey; ^2^Department of Psychiatry, Midyat State Hospital, Mardin, Turkey; ^3^Department of Psychiatry, Bingöl State Hospital, Bingöl, Turkey; ^4^Department of Psychiatry and Neurosciences, Charité Campus Mitte (CCM), Charité-Universitätsmedizin Berlin, Berlin, Germany; ^5^Department of Psychology, Health and Medical University, Potsdam, Germany

**Keywords:** alcohol use disorder (AUD), reversal learning, reinforcement learning, computational modeling, cognitive flexibility

## Abstract

**Background:**

Alcohol use disorder is characterized by perseverative alcohol use despite negative consequences. This hallmark feature of addiction potentially relates to impairments in behavioral flexibility, which can be measured by probabilistic reversal learning (PRL) paradigms. We here aimed to examine the cognitive mechanisms underlying impaired PRL task performance in patients with alcohol use disorder (AUDP) using computational models of reinforcement learning.

**Methods:**

Twenty-eight early abstinent AUDP and 27 healthy controls (HC) performed an extensive PRL paradigm. We compared conventional behavioral variables of choices (perseveration; correct responses) between groups. Moreover, we fitted Bayesian computational models to the task data to compare differences in latent cognitive variables including reward and punishment learning and choice consistency between groups.

**Results:**

AUDP and HC did not significantly differ with regard to direct perseveration rates after reversals. However, AUDP made overall less correct responses and specifically showed decreased win–stay behavior compared to HC. Interestingly, AUDP showed premature switching after no or little negative feedback but elevated proneness to stay when accumulation of negative feedback would make switching a more optimal option. Computational modeling revealed that AUDP compared to HC showed enhanced learning from punishment, a tendency to learn less from positive feedback and lower choice consistency.

**Conclusion:**

Our data do not support the assumption that AUDP are characterized by increased perseveration behavior. Instead our findings provide evidence that enhanced negative reinforcement and decreased non-drug-related reward learning as well as diminished choice consistency underlie dysfunctional choice behavior in AUDP.

## Introduction

Adaptive decision-making requires both learning from reward and punishment and updating reward and punishment contingencies in a changing environment. Substance use disorder (SUD) is characterized by perseverative drug use despite negative social, economic, and health consequences, which is thought to reflect cognitive inflexibility. Recent theories thus emphasize that inflexible decision-making is key to the pathophysiology of addiction (1).

Probabilistic reversal learning (PRL) paradigms, in which subjects must adapt to changes in stimulus–outcome contingencies, have been applied to examine cognitive flexibility (2). Some studies using PRL tasks have reported higher perseverative responses after reversals supporting impaired cognitive flexibility in patients with SUD (3–7). However, available studies have not revealed robust and consistent findings as some studies have failed to evidence higher perseverative responses in patients with alcohol use disorder (AUDP) (8, 9) as well as in amphetamine (3), cocaine (10), and methamphetamine (11) use disorder patients compared to controls. The reasons for the heterogeneity in these findings are unclear but may be partly due to differences in the clinical characteristics of the sample such as stage of addiction, psychiatric comorbidity, and medication. Furthermore, previous studies used different definitions to operationalize perseverative behavior which also makes the interpretation and comparison of previous findings difficult.

In addition, most previous studies have used means of behavioral task measures to examine decision-making abnormalities in SUD which might be influenced by different potential underlying mechanisms. So far, only a few previous studies have used computational models of reinforcement learning to infer about the latent cognitive mechanisms underlying impaired PRL task performance in SUD. Such computational models rely on the assumption that agents try to maximize total reward and minimize punishment in the long term by learning from positive and negative feedbacks *via* reward prediction error signals (12, 13). Essentially, such computational models allow to infer how effective subjects incorporate rewards and punishments to update their action values, thus providing a powerful approach to study impaired decision-making more mechanistically (14). For instance, by using this analytic approach, previous studies using different decision-making tasks have mostly found decreased choice consistency in patients with SUD. One previous study using the Iowa Gambling Task (IGT) (15) has found that patients with polysubstance use disorder showed less consistent choices but similar learning rates from reward and punishment compared to controls (16). Another study using the same task has reported reduced loss aversion and subtle differences in overall learning in opioid users but not in stimulant users relative to controls (17). Another recent study using the IGT has reported increased random exploration in patients with methamphetamine use disorder (18). Increased switching behavior rather than stick with decisions even if they are rewarded as well as lower learning rate from losses and an increased learning rate from gains have also been shown in patients with polysubstance use disorder (19). Another study using a probabilistic instrumental learning task has reported a decreased tendency to repeat prior responses in patients with opioid user disorder compared to controls (20). With regard to PRL task, two previous studies observed no alteration in neural encoding of reward prediction errors (21, 22). Moreover, there is no evidence for altered reward or punishment learning rates for the chosen stimulus in AUDP (21–24). However, these previous studies used PRL tasks with a comparably small number of reversals which may limit accurate estimation of the learning parameters. Indeed, a recent study using a PRL task with higher number of reversals found reduced reward learning, while increased learning from punishment (non-reward) in patients with stimulant use disorder (25) indicating the role of altered reinforcement learning in the maintenance of addiction. Taken together, findings of previous computational modeling studies suggest that increased random choices rather than perseveration seem to at least partly underlie abnormal decision-making processes in patients with SUD. However, evidence for learning from reward and punishment seems somewhat mixed in patients with SUD.

Most reversal learning tasks in humans have relied on non-drug rewards and punishments as opposed to drug rewards. There is accumulating evidence that addicted individuals show reduced responsivity to alternative rewards (26, 27). The mechanism underlying this shift provides a potential explanation why individuals with SUD find alternative, non-drug-related rewards and activities hardly rewarding. With regard to responsivity to punishment, previous studies have yielded mixed results. Some studies have shown decreased punishment sensitivity or reduced loss aversion (28–31), while others have shown increased sensitivity to punishment in SUD patients (25, 32). The former results have been interpreted as a potential mechanism underlying habitual drug intake (drug intake despite negative consequences), whereas the latter results have been interpreted as a potential mechanism underlying drug intake that is driven by negative states, such as withdrawal periods (33, 34).

In the present study, we aimed to build upon these recent reports and further elucidate the role of reward and punishment sensitivity as well as behavioral perseveration in early abstinent AUDP. We hypothesized that AUDP would show more random choices rather than perseverative responses relative to healthy controls. However, given the previous results were mixed, our investigation on learning from reward and punishment was exploratory.

## Materials and methods

### Participants

Twenty-eight inpatient male AUDP who had completed detoxification process were included in the study. All patients were free of benzodiazepines and other psychotropic medications for at least 5 days. Twenty-seven male healthy controls (HC) were matched to the patients with regard to age and education level. All subjects gave written informed consent to participate in the study, and the study was approved by the local ethics committee. As described elsewhere (35), exclusion criteria for the AUDP were as follows: (1) any lifetime substance use disorder other than alcohol (except nicotine), (2) current or past history of any serious psychiatric illness, including psychotic or bipolar disorder except for a past (but not current) history of major depressive disorder, (3) current or past history of any significant neurological disorders, (4) history of loss of consciousness for more than 30 min, and (5) any severe hepatic, endocrine, and renal diseases. HC met the same criteria as patients, except for the history of alcohol use disorder. All subjects were interviewed using the Structured Clinical Interview for DSM-IV Axis I Disorders to exclude participants with past or current comorbid Axis I diagnoses and to confirm the diagnosis of alcohol dependence in the clinical group. Michigan Alcoholism Screening Test (MAST) was used in the evaluation of severity of alcohol dependence. Craving was measured using Craving Typology Questionnaire (CTQ) (36, 37) total score. During the standard course of inpatient treatment, regular monitoring of blood and urine for the presence of alcohol, amphetamines, barbiturates, benzodiazepines, cocaine, cannabis, and opiates was performed to assure sobriety.

### Reversal learning task

**Figure 1:**
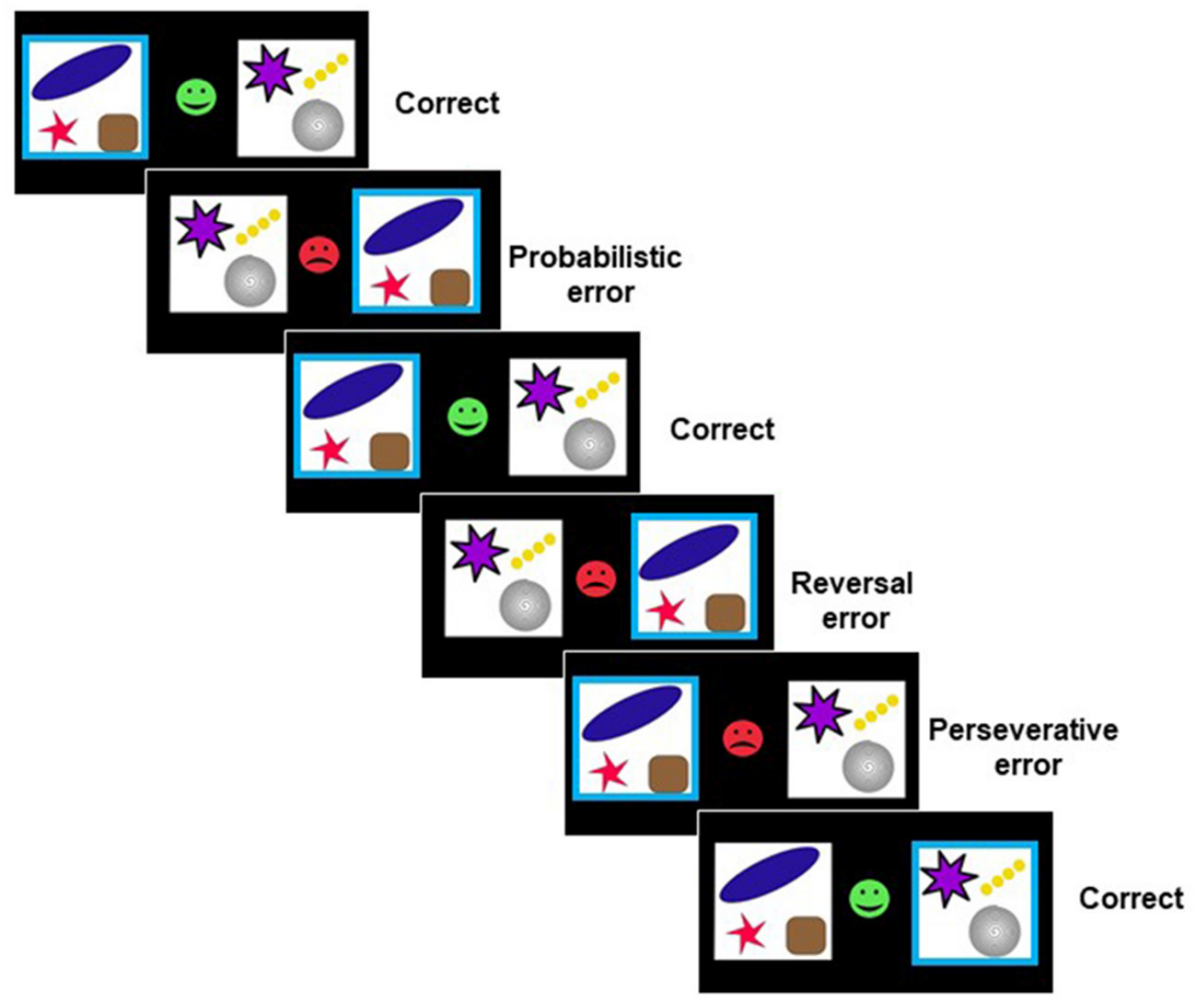
Probabilistic reversal learning task (2). On each trial, subjects were confronted with two abstract symbols. Feedback (a green smiley face or red sad face) was presented after choosing one of the symbols by a left or right button press. Using trial-and-error feedback, subjects must discover which of the two patterns is correct.

We used a probabilistic reversal learning task (2) (Figure 1) which runs in PEBL software (38). As described before (4, 39), to complete the task, participants had to finish three consecutive blocks of trials consisting of 11 discrimination stages, and, therefore, ten reversal stages. Two abstract stimuli in each block were presented simultaneously in the left and right visual fields (location randomized). There was no time limitation to produce a response in each trial. Feedback, consisting of a green smiley face for correct responses or a red sad face for incorrect responses, was presented immediately after the response. Participants were told that, according to a predefined rule, one stimulus was correct on each trial, and the other stimulus was incorrect. Participants were also instructed that the rule deciding the correct stimulus would change at various points throughout the task, and they should change their response when they were confident that the rule had changed. Reversal of the stimulus–reward contingency occurred after between 10 and 15 total correct responses (including probabilistic errors: misleading feedback provided to the usually correct and rewarded response). The number of probabilistic errors between each reversal varied from 0 to 4 in a pseudorandomized sequence. Participants were given a full block of practice trials.

### Task data analyses

#### Behavioral analysis of choice behavior

We first compared conventional behavioral measures between groups including the number of correct responses and perseverative errors. Correct choices were defined as selecting the stimulus with the higher reward probability. Following previous reports (5), perseverative errors were defined as selecting the previously rewarded stimulus at least once following a loss after a reversal. The perseverative error rate was calculated by dividing the number of perseverative errors by the number of sequences on which criterion for perseveration was met (5). To meet criterion for perseveration, participants had to make at least one consecutive response to the previously rewarded stimulus immediately following reversal, excluding the reversal error itself. Correct choices and perseverative errors were compared between groups using independent *t*-tests.

We then analyzed switching behavior as a function of the outcome in the preceding trial by using mixed-effects logistic regression. More precisely, we used a binomial link function to regress stay/switch behavior on the previous trial outcome (fixed effect: reward/no reward coded as 0.5 and −0.5) and group (fixed effect HC/AUDP coded as 0.5 and −0.5). Subjects were added as random effects (random intercept model). As we were particularly interested in whether groups showed different stay/switch behavior after rewarded and unrewarded trials, we tested for interaction between group and outcome.

As noted by Perandres-Gomez et al. (40), the abovementioned analysis of stay/switch behavior presents some interpretation problems, as stay/switch behavior does not exclusively depend on the previous trial, but on the accumulation of outcomes for previous stay/switch responses. To disentangle this, we performed a third line of analyses where we calculated the number of consecutive stay responses preceding the present trial, in the presence of cumulative negative feedback. In line with Perandres-Gomez et al. (40), cumulative negative feedback was expressed on a 0–3 scale, where 0 stands for a positive feedback on the last trial; one stands for a single negative feedback in the last trial; two stands for two consecutive negative feedbacks in the last two trials with one stay response in the previous trial; and three stands for three consecutive negative feedbacks in the last three trials with two consecutive stay responses in the previous two trials. In this analysis, we regress stay/switch behavior on cumulative negative feedback and group and tested for interaction. Again, subjects were added as random effects (random intercept model).

Regression analysis was conducted using generalized linear mixed-effects models implemented with the lme4 package (41) in the R programming language, version 3.1.2 (cran.us.r-project.org). *Post-hoc* comparisons were analyzed by pairwise contrasts using the lsmeans package (42) with Tukey's method for multiple comparisons.

#### Computational modeling

We fitted two reinforcement learning models to trial-by-trial choice data of the PRL task using hierarchical Bayesian analysis separately for each group using the R package hBayesDM (43). The hierarchical Bayesian approach assumes that individual parameters are drawn from group-level normal distributions. Normal (mean = 0, sd = 1) and half-Cauchy (location = 0, scale = 5) distributions were used for the priors of the group-level normal means and standard deviations, respectively (43). Weakly informative priors were employed to minimize the influence of those priors on the posterior distributions when the sample sizes are small (43). The hBayesDM package applies inverse probit transformation for parameters that are bounded between 0 and 1 (e.g., learning rate) to convert the unconstrained values into this range (43). In addition, hBayesDM package transforms parameters which bounded between 0 and +∞ (e.g., inverse softmax temperature) to a [0, upper limit] range by multiplying its inverse probit transformed values by upper limit (43). The first model was the reward-punishment model (RP) with separate learning rates for reward and punishment (44, 45). This RP model has three parameters, a_rew_ and a_pun_, which represent the speed of learning from positive and negative feedback, respectively, and inverse temperature (β) indicating decision variability (choosing the best option more consistently) (44). β could range between 0 and +∞. Lower values of β represent more random choice and lower sensitivity to the value of outcomes. Reduced learning from punishment would underlie perseverative behavior according to the RP model. The second model was an experience-weighted attraction (EWA) model (46). This EWA model has three parameters, learning rate (a) represents speed of learning from feedback; β; and an experience decay factor (ρ) indicating the impact of past experience with respect to incoming information (44). Increases in experience decay factor might underlie perseverative behaviors according to the EWA model. Markov chain Monte Carlo (MCMC) simulations by drawing 20,000 samples and burning the first 2,000 were used to generate posterior distribution of group-level model parameters, while accounting for individual differences. Convergence of the MCMC was assessed by both visual inspection of the Markov chains and computing the R-hat Gelman-Rubin statistics where successful coverage is indicated by values close to 1 (47). Leave-one-out information criterion (LOOIC) was used for model selection (lower values indicate better model fit) (48). To compare the parameters from the winning model between two groups, we calculated the 95% highest density interval (HDI) of the differences between each group parameter. A parameter was considered to significantly differ between groups if the HDI did not overlap 0 (17, 43).

## Results

Table 1 shows the demographic and alcohol use variables for the groups.

**Table 1:** Demographic and clinical characteristics of AUDP and healthy control subjects.

	**AUDP (*n* = 28)**	**Controls (*n* = 27)**	**Statistic**	***P*-Value**
Age (years)	39.3 ± 6.6	38.4 ± 6.5	*t* = 0.477	0.636
Education (years)	11.1 ± 4.1	11.6 ± 3.8	*t* = −0.490	0.626
Age at first use (years)	16.2 ± 4.8			
Duration of regular use (years)	14.7 ± 8.9			
Duration of abstinence (days)	26.8 ± 14.2			
MAST score	20.6 ± 5.7			
CTQ score	69.1 ± 15.0			

Data are presented as mean +/– standard deviation. AUDP, alcohol use disorder patients; MAST, Michigan alcoholism screening test; CTQ, craving typology questionnaire.

### Choice behavior

**Figure 2:**
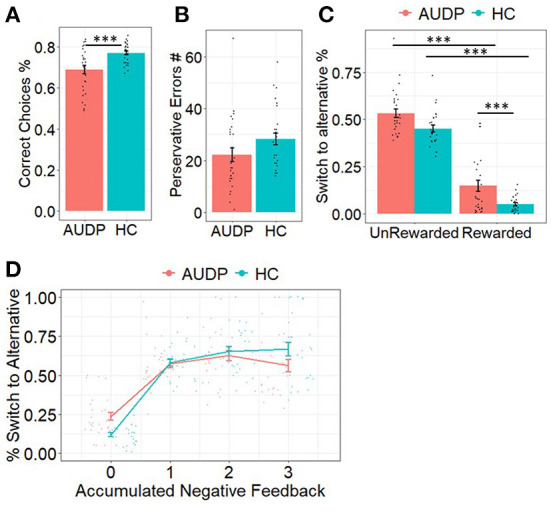
Results of the choice analyses. **(A)** AUDP made significantly less correct responses. **(B)** Perseverative errors were similar between groups. **(C)** AUDP showed decreased win–stay behavior compared to HC. **(D)** AUDP showed increased switching behavior after no or limited negative feedback but decreased switching after multiple negative feedback compared to HC. ***p < 0.001.

HC made significantly more correct responses than AUDP [*t*_(53)_ = −3.54, *p* = 0.001; Figure 2A]. Both groups did not significantly differ on the overall amount of perseverative errors (Figure 2B, Table 2). Moreover, perseverative error rates did not significantly differ between groups (Table 2).

**Table 2:** Conventional behavioral measures of AUDP and healthy control subjects.

	**AUDP (*n* = 28)**	**Controls (*n* = 27)**	**Statistic**	***P*-Value**
Perseverative errors	22.25 ± 14.05	28.3 ± 11.96	*t* = −1.72	0.091
Perseverative error rate	1.74 ± 0.52	1.56 ± 0.46	*t* = 1.31	0.198

Data are presented as mean +/– standard deviation. AUDP, alcohol use disorder patients.

Analyses of switching behavior as a function of outcome of the previous trial indicated a significant main effect of reward (Estimate_outcome_ = 0.27, SE = 0.06, *p* < 0.001), suggesting that rewarded trials were more likely to be repeated than punished trials. There was also a significant main effect of group (Estimate_group_ = 0.67, SE = 0.11, *p* < 0.001). *Post-hoc* analyses indicated that the HC repeated the previous choice more often than the AUDP. We also found a significant outcome x group interaction on repetition probability (Estimate_outcomexgroup_ = 0.96, SE = 0.07, *p* < 0.001). *Post-hoc* comparisons revealed that the AUDP repeated the previous choice less likely following rewarded trials (Estimate_AUDPreward − HCreward_ = −1.15, SE = 0.12, *p* < 0.001). There was no difference for punished trials (Estimate_ADPpunish − HCpunish_ = −0.19, SE = 0.11, *p*= 0.350) suggesting that the AUDP showed significantly less win–stay and similar lose–shift behavior relative to the HC (Figure 2C).

Our third analysis was conducted to see whether cumulative negative feedback resulted in altered switch behavior in AUDP compared to HC. In line with Perandres-Gomez et al. (40), we calculated a score for cumulative negative feedback ranging from 0 to 3 indicating how much negative feedback the individual had experienced in the trials before. Again, this analysis indicated a significant main effect of group (Estimate_group_ = 0.76, SE = 0.12, *p* < 0.0001), suggesting that AUDP showed generally more switching behavior. Moreover, the analysis revealed a significant main effect of accumulated negative feedback (Estimate_accneg_ = −0.88, SE = 0.02, *p* < 0.0001), indicating that participants were more likely to switch with the accumulation of negative feedback. Furthermore, we found a significant interaction between group and accumulated negative feedback (Estimate_groupxaccneg_ = −0.69, SE = 0.04, *p* < 0.0001). As Figure 2D reveals, the different slopes for the two groups suggest a mixture of premature switching (instability) and perseveration in AUDP. This is reflected in an elevated proneness to switch with no or little negative feedback and a slightly elevated proneness to stay when accumulation of negative feedback would make switching a more optimal option.

### Computational models

**Figure 3:**
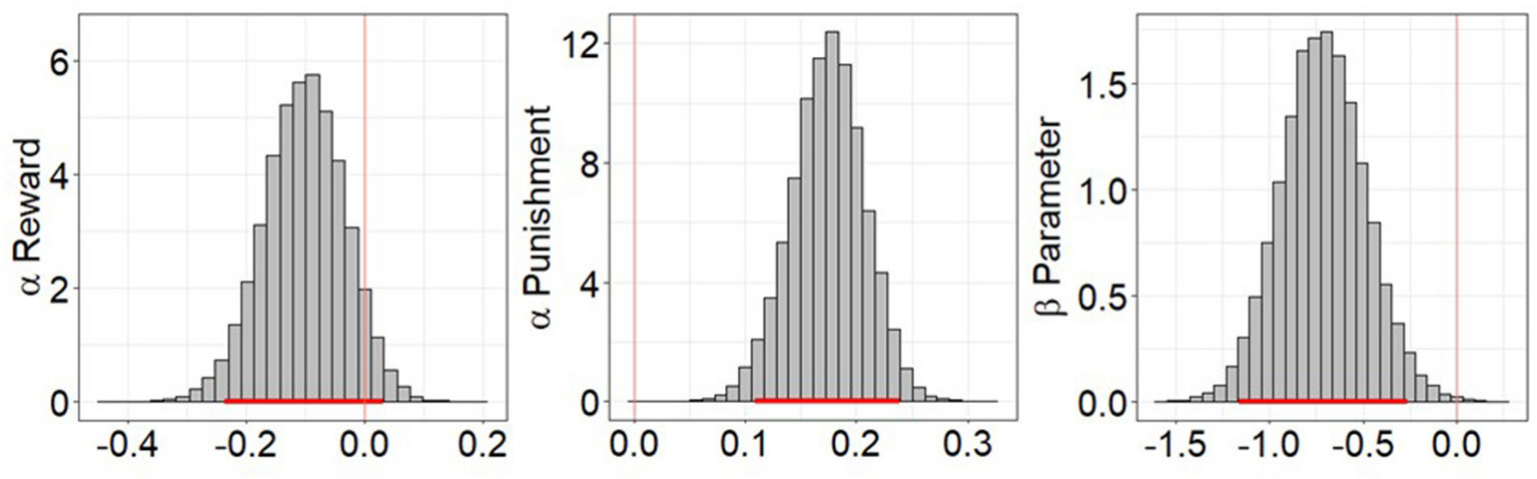
Posterior distribution differences of reward-punishment model parameters between alcohol use disorder patients (AUDP) and healthy controls (HC). A parameter was considered to significantly differ between groups if the 95% highest density interval (HDI) did not overlap 0. Punishment learning rate was significantly higher in AUDP, but reward learning rate was only slightly lower in the AUDP than HC. AUDP also showed significantly lower inverse temperature than HC, suggesting reduced reward sensitivity. The red bar indicates the 95% HDI of the group differences.

LOOIC scores were lower for RP model (AUDP = 17,667.22, HC = 11,157.05) than the EWA model (AUDP = 17,706.96, HC = 11,193.27) in both groups. Comparison of the posterior distributions of parameters from the winning RP model between groups indicated that the learning from negative feedback (a_pun_) values were significantly higher in the AUDP than HC (95% HDI = 0.109–0.239). Conversely, the learning from positive feedback (a_rew_) values were slightly lower in the AUDP than HC (95% HDI = −0.236–0.033). The β values were also significantly lower in the AUDP than HC (95% HDI = −1.161 to −0.265), suggesting more random choice and lower value sensitivity (Figure 3). All parameters had R-hat values between 0.99 and 1.01. There were no divergent transitions after warmup in any of the models and samples, with the exception of 1 divergent transition in the EWA model for the HC group.

In AUDP, Spearman's correlation analysis showed that addiction severity measured by the MAST, craving measured by the CTQ total score, duration of regular use, and duration of abstinence were not related to posterior means of α_pun_, α_rew_, or β parameters. β parameters were related positively with α_pun_ (rho = 0.418, *p* = 0.028) but not with α_rew_ (rho = 0.048, *p* = 0.810) within AUDP. In HC group, there were no correlations between β parameters with α_pun_ (rho = −0.356, *p* = 0.068) and α_rew_ (rho = −0.065, *p* = 0.746).

We performed parameter recovery for the winning RP model for the HC and for the AUDP groups. To this end, we performed posterior predictive simulations of the models. For each posterior sample, we simulated (posterior predictive) choices on the task. The choice that was most often simulated for a given subject and trial was chosen as the simulated choice. To these simulated choices, we fitted the RP model. We extracted the mean parameter values based on the original model fit to the empirical data (i.e., true parameter value) and compared this to the posterior means from the recovery model (i.e., simulated choices; recovered parameter). Supplementary Table 1 shows correlations, and Supplementary Figure 1 shows scatter plots for the true and recovered by-subject parameter estimates. The results showed that in both samples (AUDP and HC), recovery was good for α_pun_ and β parameter. Recovery was not optimal for the α_rew_ for HC.

We also performed posterior predictive checks for the RP model for AUDP and HC, where we simulated posterior predictive choices based on the fitted models. For each subject and trial, we computed the choice probabilities across all posterior samples. We then performed the same analyses as reported in Figure 2, but now using the posterior predictive choice probabilities instead of the empirical choices. The results from this analysis are shown in Supplementary Figure 2. They show that the model reproduces similar trends for the perseverative behavior (Figure 2B) and win–stay and lose–shift behavior (Figure 2C) but not for the lower correct choices in AUDP (Figure 2A) and decreased switching behavior after multiple negative feedback in AUDP (Figure 2D).

These results suggest that caution should be taken when interpreting results from our computational model.

## Discussion

In the present study, we aimed to investigate behavioral flexibility and its underlying latent cognitive mechanisms in AUDP using a reversal learning task. We found general impairments in learning and decision-making as reflected by lower rates of correct responses and decreased win–stay behavior in AUDP compared to HC. However, in our primary analyses, we did not find evidence for increased perseveration rates after reversals in AUDP compared with HC. We found that AUDP compared to HC showed increased switching behavior after no or limited negative feedback but decreased switching after multiple negative feedback. Our computational analysis revealed enhanced learning from negative feedback and a tendency to reduced learning from positive feedback in AUDP. Moreover, we found lower β values in the AUDP, suggesting that AUDP show more random behavior and/ or less sensitivity to the value of outcomes. These findings highlight the benefits of reinforcement learning models to provide a mechanistic understanding of impaired decision-making in AUDP.

Several rodent studies have shown that excessive alcohol intake renders individuals prone to habitual responding that is characterized by repetitions of actions despite outcome devaluation (49, 50). Such habitual response tendencies have been argued to underlie ongoing alcohol intake despite negative outcomes in AUDP (51, 52). However, in humans, evidence for this assumption is mixed (34, 53, 54). In the reversal learning paradigm, increases in perseverative responding after reversals or decreases in punishment learning rates potentially reflect habitual response tendencies. In the present study, we found no evidence for such habitual response tendencies in AUDP, which mirrors findings of some studies that have reported no perseverative behavior after reversals in patients with alcohol (8), opiate and amphetamine (3), and methamphetamine (11) use disorder. Our analyses where we tested how accumulated negative feedback impacted switch behavior indicated that AUDP showed increased switching behavior particularly after limited negative feedback. Likewise, our computational analyses indicated enhanced learning from negative feedback in AUDP, which is in line with models emphasizing the importance of negative reinforcement in the maintenance of addiction (26, 27, 34, 55, 56). Hogarth (34) recently argued that instead of habitual responding, increased goal-directed action selection under negative affect might underlie ongoing alcohol intake despite negative outcomes in AUDP. According to this, aversive states, such as withdrawal powerfully increases the expected alcohol value leading to alcohol intake which momentarily outweighs the expected value of abstinence. This hypothesis is in line with assumptions regarding alcohol craving elicited by (expected) relief from withdrawal and associated negative mood states (57). Although in our study, we did not assess goal-directed action selection directly, our finding of increased punishment sensitivity in AUDP aligns well with the hypothesis that actions in AUDP are excessively driven by negative states.

In contrast to our findings, two previous studies using PRL tasks in AUDP have found no differences in punishment learning rates for the chosen stimulus although their behavioral analysis revealed similar findings such as lower win–stay but similar lose–shift choices in AUDP (23, 24). Inconsistencies between our and these studies may be due to task discrepancies with regard to the number of reversals [five and ~10 reversals in the studies by Reiter et al. (24) and Beylergil et al. (23), respectively, vs. 30 reversals in our study] which may limit accurate estimation of learning parameters. Another study which used a similar number of reversals as our study has reported increased learning from non-reward in patients with stimulant use disorder (25). Although non-rewards are functionally different from punishments, this result aligns well with our finding indicating that addicted individuals might be particularly impaired in integrating information of non-rewarding valence in their choices. Furthermore, enhanced learning from punishment and increased loss avoidance have also been found in recently abstinent patients with nicotine (32, 58) and cocaine dependence (33). Although not measured directly in the current study, our finding might be related to changes in dopamine system over the course of addiction. Prior studies have shown that long-term drug use is related to lower tonic ventral striatal (VS) (including nucleus accumbens) dopamine levels (59) which have been linked to enhanced learning from negative outcomes (60). In sum, our research extends the findings of previous studies supporting the importance of negative reinforcement in nicotine and stimulant use disorder to alcohol use disorder.

Positive reinforcement effect of drugs *via* phasic release of dopamine in VS is thought to play a key role during the initial phase of drug use (26). However, with the continued drug use, blunted phasic dopamine release and lower BOLD response within the VS to both drug-related and non-drug-related rewards has been shown in individuals with substance use disorder (59, 61, 62). Previous studies have also found reduced responsivity to monetary rewards in AUDP (63, 64). Consistent with these findings, we found that AUDP had lower win–stay responses and slightly diminished learning from positive feedback compared to HC suggesting dysfunctional reward learning. In line with our findings, previous studies using computational analysis found diminished learning from positive feedback in stimulant (25) and nicotine dependence (32). However, we have to note that whether hyporesponsivity to rewards is a result of extended drug use or a pre-existing vulnerability trait is an ongoing debate (65). We are unable to establish a clear temporal relationship between hyporesponsivity to rewards and AUDP, due to the cross-sectional design of our study. Some longitudinal studies suggest delayed recovery of dopamine D2-receptor function following detoxification being associated with poor treatment outcome (66–68), so longitudinal studies should assess reward sensitivity and the putative neurobiological correlates during alcohol intake, detoxification, and abstinence.

AUDP showed more random choices as evidenced by lower β values than HC. Our regression analyses indicated increased switching behavior particularly in light of positive reinforcement. Moreover, further analyses indicated that switching behavior in AUDP was also increased after none or limited negative feedback, whereas after multiple negative feedback, AUDP showed a tendency to switch less. Our findings converge with two previous studies using computational analyses that reported excessive switching behavior in cocaine (10) and nicotine (69) use disorder patients. In addition, these results could be interpreted based on the active inference framework of addiction (70). According to this framework, substance use leads to suboptimal precision which reflects the degree of stochasticity or goal-directedness of behavior (71). Therefore, our finding of more random choices in AUDP is in line with previous studies that have reported lower action precision in substance use disorder (19, 71). Crucially, the task used in this study does not enable to functionally dissociate randomness from exploration. Interestingly, some previous studies have suggested that tonic dopamine might either increase (72) or decrease exploration (73). As AUDP show reduced availability of D2 receptors (59, 74, 75), putatively reflecting a downregulation of the dopaminergic system, future studies should apply appropriate tasks to further investigate the link between dopaminergic functioning and the exploration/exploitation trade-off in AUDP.

The findings of this study should be interpreted after consideration of the following limitations. An important limitation of this study is its cross-sectional nature. Thus, it is unclear whether our findings resulted from detrimental effects of chronic alcohol use on the brain or pre-existed in individuals more prone to alcohol use. Longitudinal studies are needed to clarify this issue. Another limitation of the study is that it included only males, thus limiting generalizability. Third, although AUDP were not clinically depressed, symptom severity was not assessed with a scale and thus we may have underestimated subclinical symptoms. Furthermore, we did not exclude AUDP with a history of major depressive disorder as the differential diagnosis between alcohol-induced depressive disorder and independent major depressive disorder is challenging. All AUDP except three reported a history of lifetime major depressive episode. Given that major depressive disorder has been previously shown to affect performance on probabilistic reversal learning tasks (76), inclusion of AUDP with a history of major depressive disorder might contribute to at least some of these observed differences. Fourth, we did not examine other cognitive measures such as working memory which might impact our results. In addition, because we used the hBayesDM package, our models did not include a perseveration parameter which might impact our results regarding the learning rates and inverse temperature. Sixth, because of poor parameter recovery of model parameters particularly learning rates for rewards and given the RP model did not well-captured some behaviors, our findings should be interpreted cautiously.

In conclusion, our findings provide further evidence for the importance of higher negative reinforcement and lower reward sensitivity in AUDP particularly during early abstinence.

## Data availability statement

The raw data supporting the conclusions of this article will be made available by the authors, without undue reservation.

## Ethics statement

The studies involving human participants were reviewed and approved by Department of Psychiatry, Katip Celebi University Ataturk Education and Research Hospital, Izmir, Turkey. The patients/participants provided their written informed consent to participate in this study.

## Author contributions

BB, NZ, and MS conceptualized and designed the study. BB, SI, AB, SD, and GB were involved in planning and managing the data collection. NZ, MS, DS, and AH were involved in the statistical analysis. NZ wrote the first draft of the manuscript. All authors contributed to and have approved the final manuscript.

## Funding

This work was funded in part by the Deutsche Forschungsgemeinschaft (DFG, German Research Foundation)—Collaborative Research Grant, SFB Project-ID 402170461—TRR 265 [Heinz et al. (77), (to AH and MS)], and Excellence Strategy—EXC-2049—390688087 (to AH).

## Conflict of interest

The authors declare that the research was conducted in the absence of any commercial or financial relationships that could be construed as a potential conflict of interest.

## Publisher's note

All claims expressed in this article are solely those of the authors and do not necessarily represent those of their affiliated organizations, or those of the publisher, the editors and the reviewers. Any product that may be evaluated in this article, or claim that may be made by its manufacturer, is not guaranteed or endorsed by the publisher.
