# Supplementary material for: Computational analysis of probabilistic reversal learning deficits in male subjects with alcohol use disorder
Source: Front Psychiatry. 2022 Oct 19;13:960238. doi: 10.3389/fpsyt.2022.960238 (PMC9626515; doi:10.3389/fpsyt.2022.960238)
Supplement: Supplementary file 1 [file Data_Sheet_1.PDF]

Supplementary Information

Supplementary Table 1. Pearson correlations between true and recovered parameters from the RP model for HC and AUDP subjects.

	Arew	Apun	Temperature
AUDP	0.39	0.62	0.72
HC	0.17	0.62	0.37

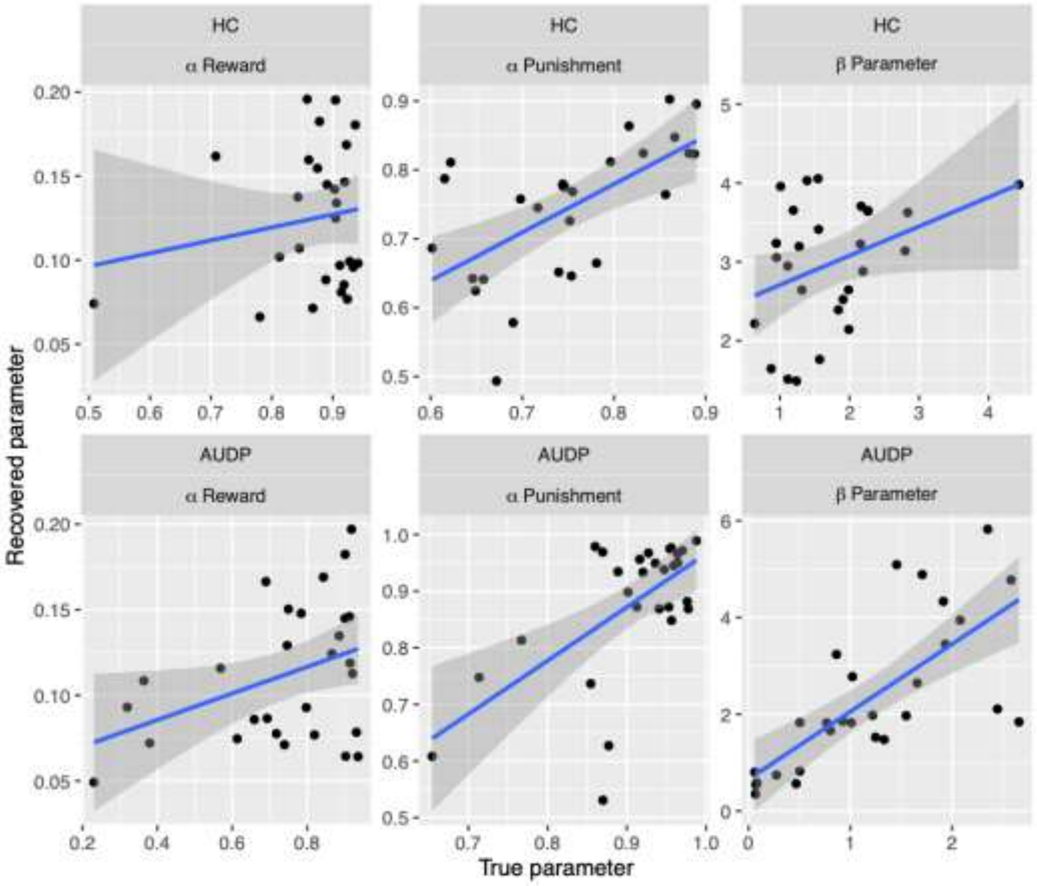

Supplementary Figure 1. Scatter plots together with predictions from linear regressions for true and recovered parameters from the RP model for HC and AUDP subjects.

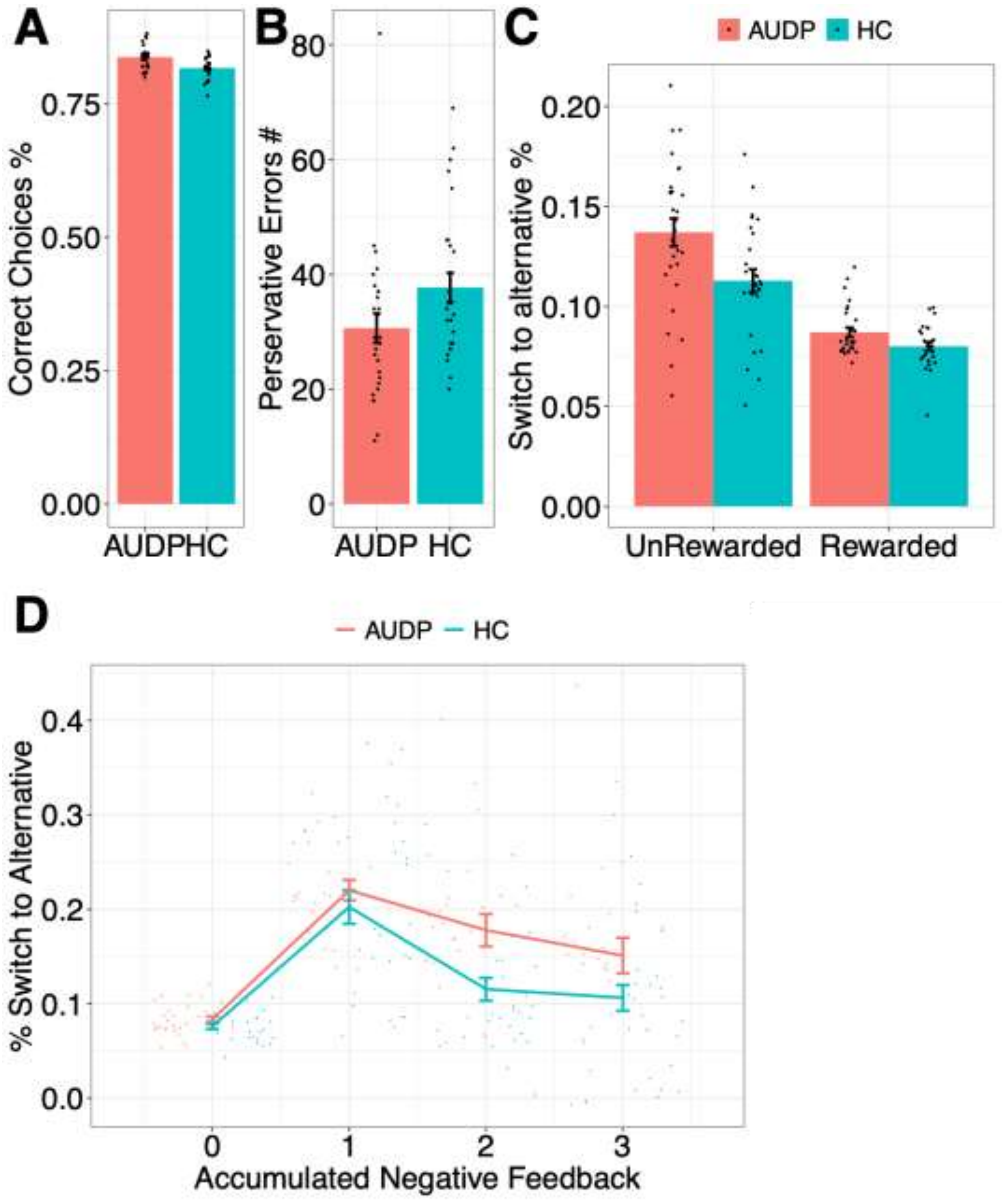

Supplementary Figure 2.: Results of the posterior predictive choices based on the winning model. (A) correct responses. (B) Perseverative errors (C) win-stay behavior. (D) switching behavior after multiple negative feedback
